# Development and validation of a machine learning model using electronic health records to predict trauma- and stressor-related psychiatric disorders after hospitalization with sepsis

**DOI:** 10.1038/s41398-023-02699-6

**Published:** 2023-12-18

**Authors:** Santiago Papini, Esti Iturralde, Yun Lu, John D. Greene, Fernando Barreda, Stacy A. Sterling, Vincent X. Liu

**Affiliations:** 1 Division of Research, Kaiser Permanente Northern California, Oakland, CA USA; 2 Department of Psychology, University of Hawaiʻi at Mānoa, Honolulu, HI USA

**Keywords:** Psychiatric disorders, Prognostic markers

## Abstract

A significant minority of individuals develop trauma- and stressor-related disorders (TSRD) after surviving sepsis, a life-threatening immune response to infections. Accurate prediction of risk for TSRD can facilitate targeted early intervention strategies, but many existing models rely on research measures that are impractical to incorporate to standard emergency department workflows. To increase the feasibility of implementation, we developed models that predict TSRD in the year after survival from sepsis using only electronic health records from the hospitalization (*n* = 217,122 hospitalizations from 2012-2015). The optimal model was evaluated in a temporally independent prospective test sample (*n* = 128,783 hospitalizations from 2016-2017), where patients in the highest-risk decile accounted for nearly one-third of TSRD cases. Our approach demonstrates that risk for TSRD after sepsis can be stratified without additional assessment burden on clinicians and patients, which increases the likelihood of model implementation in hospital settings.

Every year millions of patients develop sepsis, a life-threatening, dysregulated immune response to infection [1]. Advances in sepsis care over the prior two decades have improved survival rates, thus requiring an increased focus on the prevention of adverse long-term mental health outcomes, including posttraumatic stress disorder (PTSD) and other trauma-related psychiatric outcomes [1]. A systematic review [2] documented a wide range of PTSD prevalence among survivors of sepsis and other conditions treated at intensive care units (8% to 51%). Accurate and efficient prediction of PTSD risk may facilitate targeted screening and delivery of preventive and early interventions after hospital discharge [3]. While prior PTSD research typically focused on the identification of key risk factors [4], machine learning models aim to increase predictive accuracy by incorporating a large set of individually weak predictors and complex interactions [3]. Among individuals treated at intensive care units, systematic reviews document several predictors of PTSD, including pre-existing psychopathology, benzodiazepine administration, female sex, age, sedation, and trait anxiety [2, 5]. However, machine learning models that could potentially incorporate these and other variables to predict trauma-related psychiatric outcomes, including PTSD after survival from sepsis have not been developed.

Recent reviews summarize research on machine learning models to predict PTSD after a variety of potentially traumatic experiences, including military combat, natural disasters, transportation accidents, physical and sexual assault, and concussion [3, 6]. Some of these studies demonstrated that machine learning models could accurately identify the emergency department (ED) admits at high risk for PTSD after a variety of injuries resulting from motor vehicle accidents, falls, work-related accidents, physical and sexual assault, gunshot wounds, and terrorist attacks [7–12]. However, several limitations may impede real-world implementation. Most of the existing models have been developed from extensive research assessment batteries that are not part of standard ED workflows. Although some of these models included information that can be found in patient charts, including patient characteristics (e.g., age, sex, body mass index), injury characteristics, vital signs, lab results, medications, and pre-existing diagnoses, many of the most important predictors were from study surveys that assessed acute psychological responses, social support, and resilience [7–12]. Another limitation of past studies is that models were developed with relatively small samples ranging from 152 to 1003 participants [7–12]. Moreover, many of the samples were substantially reduced as a result of stringent inclusion/exclusion criteria, which may limit the generalizability of models to patient populations that meet similar research criteria. Finally, most studies relied on self-reported symptom measures to assess PTSD outcomes and did not consider other trauma- and stressor-related disorders. Our aim was to overcome these limitations by developing and validating a predictive model that relied solely on electronic health records (EHR) data collected at the time of hospitalization to predict clinician-diagnosed trauma- and stressor-related disorders (TSRD) in the year post-discharge from hospitalization for sepsis in a large, multi-site sample that included all survivors.

## Methods

We used the Transparent reporting of a multivariable prediction model for individual prognosis or diagnosis (TRIPOD) guidelines (https://www.equator-network.org/reporting-guidelines/tripod-statement/). The TRIPOD Checklist for Prediction Model Development and Validation is provided in the Supplementary Information (Appendix 1). This study was approved by the Kaiser Permanente Northern California Institutional Review Board. The data from this study cannot be shared publicly as we do not have permission from patients to share their data outside of the Kaiser Permanente Northern California healthcare system.

### Study Sample

**Fig. 1:**
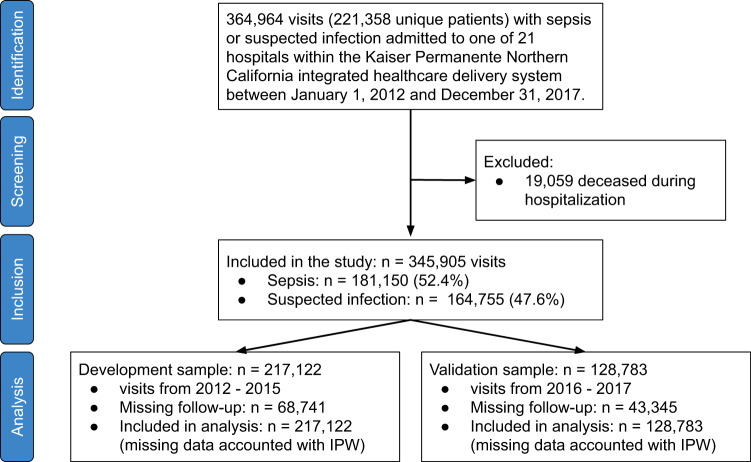
Study data flow. IPW = inverse probability weighting.

Inclusion criteria were hospitalizations for sepsis or suspected infection that were admitted through emergency departments across 21 hospitals within the Kaiser Permanente Northern California integrated healthcare delivery system between January 1, 2012, and December 31, 2017 (Fig. 1). This resulted in the inclusion of 364,964 hospitalizations for sepsis or suspected infection (221,358 unique patients). We excluded 19,059 cases (5.2%) in which the patient died during the hospitalization. No other exclusion criteria were applied, resulting in a final sample of 345,905 hospitalizations (210,946 unique patients).

### Predictors

To extract predictors, we used EHR data collected during the hospitalization, including the types of variables that have been used in prior studies that developed predictive models of psychiatric outcomes after hospitalization (e.g., patient characteristics, vital signs, lab results, medications, and pre-existing psychiatric diagnoses) [7–12]. Additional variables were selected based on clinical considerations of sepsis-related factors that could potentially contribute to long-term medical outcomes, including cardiovascular disease [13–15]. We also used natural language processing to extract predictors from clinician notes. Across all data types, one-hot encoding was applied to categorical variables (i.e., each category was converted to a binary indicator where 1 = present, 0 = absent), and variables with near-zero variance were excluded. This resulted in a final set of 607 predictors, including medications, clinician notes, laboratory values, mental status assessments, admission and hospitalization information, vital signs, demographics, and mental health diagnoses; data preparation procedures for each of these data types are described below.

Two-hundred and forty-six of the predictors (40.5%) were derived from medications that were administered during hospitalizations. For each medication, several indicators were created that varied in their level of specificity: 1) therapeutic class, 2) pharmaceutical class, 3) pharmaceutical subclass, and 4) National Drug Code (NDC; the Food and Drug Administration’s drug identifier). For example, the administration of olanzapine resulted in indicators for 1) psychotherapeutic drugs (therapeutic class), 2) atypical antipsychotic, dopamine-serotonin antagonist (pharmaceutical class), 3) thienobenzodiazepine (pharmaceutical subclass), and 4) 00002411633 (NDC). Each of the medication variables captured the number of times a patient received each class of (or specific) medication during the hospitalization. For example, a value of 3 for the variable “00002411633 (NDC)” would indicate that the specific medication Olanzapine (the drug that corresponds with the NDC code 00002411633) was administered 3 times during the hospitalization, a value of 12 for the variable “Psychotherapeutic medication class” would indicate psychotherapeutic medications (which is a class that includes many specific medications) were administered 12 times, and so on.

Ninety-eight of the predictors (16.1%) were presenting symptoms from clinician notes in the first 24 hours of hospitalization. The rationale for focusing on notes from the first 24 hours of hospitalization was to capture disease severity at the onset. The I2E natural language processing (NLP) software (Linguamatics I2E 5.4.1R13, Cambridge, UK) was used to identify and process presenting symptoms from raw text based on existing medical ontologies [16]. The most frequent terms were grouped into categories based on similarity and binary variables were created for each category to indicate the presence/absence of a symptom for each hospitalization. Additional details about these NLP-derived variables are published elsewhere [16].

Seventy-five of the predictors (12.4%) were derived from laboratory values for creatinine, hematocrit, total white blood cell count, glucose, sodium, bicarbonate, blood urea nitrogen, albumin, anion gap, troponin, total serum bilirubin, lactate, arterial PaCO2, arterial PaO2, and pH. Since laboratory analyses could be repeated during a hospitalization, variables were created to indicate the first, last, minimum, maximum, and mean of each lab value.

Seventy-five of the predictors (12.4%) were derived from assessments indicating mental status, level of consciousness (e.g., alert, awake, lethargic, stuporous), orientation (e.g., person, place, time, unable to assess), Schmid assessment (e.g., periodic confusion, comatose/unresponsive), speech (e.g., clear, slurred, rambling, no verbal response), pupil response, and Glasgow Coma Scale (GCS) total. For each of these, variables were created to indicate the first and last assessment values; means, minimums, and maximums were also created for the GCS total.

Forty-six of the predictors (7.6%) were derived from admission and hospitalization information, including the admission category; Charlson Comorbidity Index; Comorbidity Point Score (COPS2); Laboratory-based Acute Physiology Score (LAPS2); height, weight, and body mass index; whether the patient was admitted to the intensive care unit (direct, late, or otherwise) and if so the length of stay; whether the patient was transported in; whether the patient was an observation-only admission; time to antibiotic administration; the principal diagnosis; and the unit for the first inpatient stay.

Thirty of the predictors (4.9%) were derived from vital signs indicating heart rate, oxygen saturation, respiratory rate, temperature, and blood pressure. Variables were created to indicate the first, last, minimum, maximum, and mean of each vital sign value.

Twenty-five of the predictors (4.1%) were derived from demographic information such as age, race, ethnicity, and sex.

Twelve of the predictors (2.0%) were derived from psychiatric diagnostic codes recorded during the hospitalization (indicating that the patient received services related to this diagnosis during the hospitalization) or present in the patient’s problem list (indicating that the pre-existing diagnosis may be a prominent concern in the patient’s ongoing health care): mood disorder, alcohol use disorder, substance use disorder, anxiety disorder, major depressive affective disorder (single episode unspecified, recurrent episode unspecified, or recurrent episode in full remission), alcohol abuse, depressive disorder not elsewhere classified, anxiety state unspecified, and trauma- and stressor-related disorder (see *Outcome* section below).

### Outcome

The target outcome was defined as the presence of a diagnostic code for a trauma- and stressor-related disorder (TSRD) in at least one encounter of any type (e.g., inpatient or outpatient) within 12 months postdischarge from the sepsis hospitalization. This outcome definition is based on the DSM-5 [17], which created TSRD as a distinct cluster that includes the diagnoses post-traumatic stress disorder (PTSD), acute stress disorder, adjustment disorder, other specified trauma- and stressor-related disorder, and unspecified trauma- and stressor-related disorder due to overlapping etiologies and symptoms [18]. Although the clinical impairment associated with acute stress disorder and adjustment disorder may be milder than what is typically observed in PTSD, meta-analyses suggest that it is not uncommon for these diagnoses to precede more severe mental health outcomes, including PTSD [19, 20]. Therefore, predicting risk for any of these disorders may facilitate the development and evaluation of preventive and early interventions, including targeted screening. Given systematic reviews and meta-analyses found long-term rates of prevalence of psychiatric outcomes among critical-illness survivors [2, 21, 22], a 12-month follow-up window was selected to capture cases that may have delayed emergence (or reporting) of symptoms. Supplementary Table 1 includes the full list of International Classification of Diseases (ICD) codes that were included in the outcome.

Among the 345,905 hospitalizations that were included in this study, 112,086 (32.4%) had censored (unobserved) outcomes in the one-year follow-up window due to either death (*n* = 11,940; 3.5%) or to lapses in health insurance coverage (*n* = 100,146; 29.0%). Previous prediction models excluded cases from the model development and evaluation if the outcome was missing [7, 9, 10, 12, 23, 24]. This is problematic because complete case analyses are only valid when data are missing completely at random (MCAR) [25], which is an unrealistically strong assumption that ignores the potential for attrition bias and can result in inaccurate performance estimates. Consistent with more recent prediction models [11, 26], we accounted for these encounters by using inverse probability of censoring weights, a robust approach for including cases when outcomes are missing at random (MAR; a less stringent assumption than MCAR); this approach leads to less biased performance estimates compared to complete-case approaches [27]. A stacked ensemble of ML algorithms (i.e., the approach used for the TSRD outcome model described in Statistical Analyses) was used to estimate the probability of having an observed outcome using all the pre-discharge predictors, and the inverse of these probabilities were used as weights in the TSRD prediction models. This non-parametric approach can capture complex high-dimension interactions among variables and does not make distributional assumptions about relations among variables and missingness. These weights were used across all analyses so that missing outcome data were always taken into account.

### Statistical analysis

All analyses were conducted with the open-source statistical software *R* (version 4.1.1) [28]. Supplementary Figure 1 provides a schematic of the modeling pipeline, which is described in detail below. To protect against overfitting, ML models are often developed on a random subset of the data (i.e., training or development sample) and tested on the held-out portion (i.e., test sample). However, random subsampling may also lead to inaccurate estimates of performance given that the development dataset may contain participants whose data were collected *after* participants used to validate the model. In the real world, models need to generalize to *future* cases. To get closer to this ideal we used a prospective validation approach that is designed to assess how the prediction model may perform in future cases. All data used to develop and compare candidate models were from hospitalizations with discharge dates between 2012 and 2015 (*n* = 217,122). The remaining data (discharge dates between 2016-2017; *n* = 128,783) were used to test the best-performing model selected in the development phase.

In real-world healthcare settings, predictive models can be embedded within EHR systems to make predictions at the encounter level; therefore, models are typically developed and tested using encounter-level data [29–31]. In large samples that cover a wide span of time, it is common for some patients to have multiple encounters. To reduce the influence of patients with multiple encounters in the model development phase, cross-validation folds were stratified such that encounters from the same patient were all contained within the same fold. The performance of the final model was evaluated in the temporally independent prospective test sample, which could contain new encounters from patients who also had encounters in the development sample; therefore, sensitivity analyses were conducted to examine the performance of the model on the sample of encounters from new patients only.

Models were developed with the *h2o* machine learning platform [32] using approaches with varying levels of complexity. The least complex model was logistic regression with elastic net regularization, which can yield a model with fewer predictors because some predictor coefficients may be reduced to zero. Next in order of complexity was a core-predictor gradient boosting machine (GBM) model, which is a non-parametric decision tree approach that can capture complex high-dimensional interactions among predictors. An information-theoretic approach [33] was used to identify a set of core predictors using 20% of the development sample and the remaining 80% of the sample was used to develop a GBM model to predict TSRD using only core predictors. The most complex model was a stacked ensemble of various decision-tree algorithms, including GBM, XGBoost, distributed random forests, and extremely randomized trees [34]. In this approach, predictions from these base-learners were integrated by a meta-learner to generate a final prediction. Across all approaches, cross-validation was used to train models by optimizing log-loss, which captures the discrepancy between predicted probabilities and true outcomes.

To select a final model, we considered log-loss (lower is better) and area under the receiver operating characteristics (ROC) curve (AUC; higher is better, and an AUC of 0.50 indicates random or chance-level performance); these performance metrics were estimated in the development sample using 10-fold cross-validation (8 folds for the core-predictor GBM since two of the folds were used to identify core predictors). Consistent with prior prediction models, patients who had a pre-existing trauma- and stressor-related psychiatric diagnoses were not excluded from the sample [11, 12, 24, 26]. Instead, a univariate generalized linear model that used pre-existing TSRD diagnosis as the sole predictor of TSRD in the follow-up phase served as an informative benchmark: *Y*_*i*_ = *β*_*0*_ + *β×(pre-existing PTSD)*_*i*_ + *ε*_*i*_ The purpose of testing whether models outperformed this benchmark is two-fold. First, it is a more rigorous benchmark than prediction better than chance [35]. Second, this benchmark model captures how much of the predictive accuracy is driven by pre-existing diagnosis. Similar benchmarks have been used in other predictive models of psychiatric outcomes after trauma exposure [11, 12, 24, 26]. Given that simpler models may be easier to implement, we also took predictor parsimony into account such that a model with fewer predictors and comparable performance would be favored.

Once an optimal model was selected, it was applied to the independent test dataset. We assessed model discrimination with an ROC curve and AUC statistic, and model calibration with a logistic calibration curve and expected calibration error (ECE). We also measured sensitivity, specificity, and positive and negative predictive value in the test sample across deciles based on the predicted risk distribution in the development sample. The mean of each performance metric was estimated with 95% confidence intervals (CI) using weighted bootstrapping (1000 replications).

In addition to these performance metrics, we examined the relative importance of predictors in the optimal model with the Shapley Additive Explanations (SHAP) approach [36]. This method produces a plot that summarizes each predictor’s impact on model predictions across values of the predictor. As such, it provides information about the predictor’s relative importance (the magnitude of its impact on the final model prediction) and potential directionality (whether high or low values of the predictor are associated with increased or decreased probability of TSRD). Another advantage of this approach is that it is model-agnostic, which means it can be applied to any model (e.g., elastic net, GBM, or stacked ensemble). These analyses used the development sample to provide insight into what the optimal model learned. Although these analyses should not be causally interpreted, they may indicate which variables were most useful for predicting the outcome.

Finally, we examined whether model implementation may potentially lead to unfair outcomes across sociodemographic categories. Algorithmic fairness is a highly active area of research and there is currently no consensus on the optimal criteria to assess fairness [37]. We estimated ROC curves and AUC statistics across the self-reported categories of race, Hispanic/Latino ethnicity, and sex. ROC curves can reveal threshold regions where differences in sensitivity and specificity may exist across subgroups, and the AUC statistic can indicate whether there may be differences in overall discrimination across all thresholds. These analyses used the test sample because their purpose is to assess whether implementation of the final model could potentially lead to unfair outcomes.

## Results

Table 1 displays basic demographic characteristics of the sample; no meaningful differences were observed across the development and test samples. The prevalence of TSRD in the year post-discharge was 7.1% (6.8% in the development sample and 7.4% in the test sample). This was considerably higher than the prevalence of pre-existing TSRD in the medical charts at the time of hospitalization (1.5% in the development sample and 1.6% in the test sample).

**Table 1:** Demographic characteristics of the development and test samples.

	Full Sample, *N* = 345,905	Development Sample, *N* = 217,122	Test Sample, *N* = 128,783
Age, Mean (SD)	67.9 (17.7)	68.0 (17.7)	67.8 (17.7)
Women, N (%)	185,258 (53.6%)	117,334 (54.0%)	67,924 (52.7%)
Hispanic or Latino, N (%)	53,116 (15.4%)	32,213 (14.8%)	20,903 (16.2%)
Race, N (%)			
American Indian or Alaska Native	2068 (0.6%)	1265 (0.6%)	803 (0.6%)
Asian	36,213 (10.5%)	21,961 (10.1%)	14,252 (11.1%)
Black or African American	34,597 (10.0%)	21,190 (9.8%)	13,407 (10.4%)
Multiracial	25,440 (7.4%)	16,807 (7.7%)	8633 (6.7%)
Native Hawaiian or Pacific Islander	1983 (0.6%)	1140 (0.5%)	843 (0.7%)
Unknown	40,145 (11.6%)	24,022 (11.1%)	16,123 (12.5%)
White	205,459 (59.4%)	130,737 (60.2%)	74,722 (58.0%)

*Note*. Sex, ethnicity, and race were self-reported.

Model performance in the development phase was best for a stacked ensemble of decision-tree algorithms (log-loss = 0.219, AUC = 0.73, 607 predictors), followed by a gradient-boosted machine (GBM) model with core predictors (log-loss = 0.219, AUC = 0.71, 29 predictors), and an elastic net model (log-loss = 0.221, AUC = 0.71, 487 predictors). All models outperformed a benchmark model that used pre-existing TSRD diagnosis as the sole predictor of TSRD in the follow-up phase (log-loss = 0.229, AUC = 0.56). Moreover, the benchmark model results suggest that simply predicting follow-up TSRD on the basis of pre-existing TSRD is only slightly better than chance prediction, whereas incorporating predictors from the hospitalization resulted in meaningful increases in predictive performance. Specifically, when compared to the benchmark model, the other models showed a 27-30% increase in AUC, and a 3.5-4.4% reduction in log-loss.

**Fig. 2:**
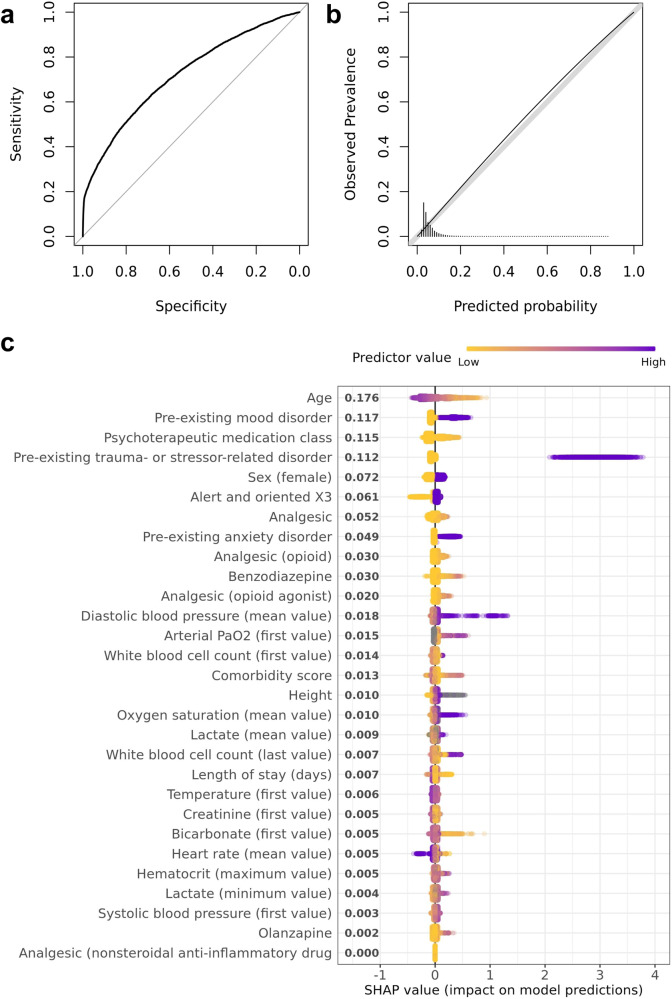
Model performance and variable importance. **a** Receiver operating characteristics (ROC) curve illustrating the tradeoff between model sensitivity and specificity based on mode-predicted probabilities of TSRD in the independent test sample. **b** Logistic calibration curve illustrating the correspondence between model-predicted probabilities and observed prevalence of TSRD in the independent test sample. **c** Shapley Additive Explanations (SHAP) plot illustrating the impact of each predictor on model predictions in the development sample. Predictors are arranged on the y-axis in order of absolute mean contribution, with exact values provided next to each predictor. Positive or negative SHAP values on the x-axis indicate higher or lower predicted probability of TSRD, respectively. In the plot, each point represents a participant in the development sample, and the color represents the value of the predictors. For example, higher values of age (blue) yielded lower predicted probabilities of TSRD, and vice versa.

We selected the GBM for validation because it achieved comparable performance to the stacked ensemble and elastic net using only 29 predictors (as opposed to hundreds). In the temporally independent prospective test sample, the GBM had similar classification performance, AUC = 0.72, 95% CI [0.71, 0.72], and low expected calibration error, ECE = 0.009, 95% CI [0.008, 0.011], suggesting good generalization (Fig. 2). Sensitivity analyses showed that results were similar after removing encounters in the test dataset from patients who also had encounters in the development dataset (AUC = 0.72, 95% CI [0.71, 0.73], ECE = 0.009, 95% CI [0.007, 0.011]; Supplementary Fig. 2). Table 2 provides the threshold-dependent metrics of the GBM model in the test sample across deciles based on the predicted risk distribution in the development sample. The 8.7% of patients whose predicted risk for TSRD fell in the highest risk decile accounted for nearly one third of the TSRD cases (Table 2).

**Table 2:** Threshold-dependent metrics of the GBM model in the test sample across deciles based on the predicted risk distribution in the development sample.

Risk Decile	Encounters within decile, % [95% CI]	Cumulative encounters, % [95% CI]	Cumulative Sensitivity, mean [95% CI]	Cumulative Specificity, mean [95% CI]	Cumulative PPV, mean [95% CI]	Cumulative NPV, mean [95% CI]
1	8.7 [8.5, 8.8]	8.7 [8.5, 8.8]	31.2 [30.1, 32.3]	93.1 [93.0, 93.3]	26.7 [25.7, 27.7]	94.4 [94.2, 94.6]
2	9.0 [8.8, 9.2]	17.6 [17.4, 17.9]	45.3 [44.0, 46.5]	84.6 [84.3, 84.8]	19.1 [18.5, 19.7]	95.1 [94.9, 95.2]
3	9.6 [9.4, 9.8]	27.2 [26.9, 27.5]	55.8 [54.5, 57.1]	75.1 [74.8, 75.4]	15.2 [14.8, 15.7]	95.5 [95.3, 95.7]
4	9.1 [8.9, 9.3]	36.4 [36.0, 36.7]	64.3 [63.1, 65.6]	65.9 [65.6, 66.2]	13.1 [12.8, 13.5]	95.8 [95.7, 96.0]
5	10 [9.8, 10.2]	46.3 [46.0, 46.7]	72.6 [71.6, 73.8]	55.8 [55.4, 56.1]	11.6 [11.3, 11.9]	96.2 [96.0, 96.4]
6	9.7 [9.5, 9.9]	56 [55.7, 56.4]	79.7 [78.6, 80.7]	45.9 [45.5, 46.2]	10.6 [10.3, 10.8]	96.6 [96.4, 96.8]
7	10.3 [10.1, 10.5]	66.3 [66.0, 66.6]	86.1 [85.2, 86.9]	35.2 [34.9, 35.6]	9.6 [9.4, 9.9]	96.9 [96.7, 97.1]
8	10.4 [10.2, 10.7]	76.8 [76.5, 77.0]	91.7 [91.0, 92.4]	24.4 [24.1, 24.7]	8.9 [8.6, 9.1]	97.4 [97.1, 97.6]
9	10.9 [10.7, 11.1]	87.6 [87.4, 87.9]	96.5 [96.0, 96.9]	13.1 [12.8, 13.3]	8.2 [8.0, 8.4]	97.9 [97.6, 98.1]
10	12.3 [12.1, 12.6]	100 [100, 100]	100 [100, 100]	0 [0, 0]	7.4 [7.3, 7.6]	NA

*Note*. *PPV* positive predictive value, *NPV* negative predictive value.

Fig. 2C illustrates the relative importance of the 29 core predictors included in the final GBM arranged in order of the magnitude of their total contribution to predictions. Each point in the SHAP plot represents the impact of that predictor on an individual’s prediction, with positive and negative values denoting increases and decreases in the probability of TSRD, respectively. A key finding from the SHAP analyses is that while pre-existing TSRD made the strongest contribution to *increased* probability of TSRD, it did not make the strongest contribution overall. This is because only a small fraction of the development sample (1.5%) had a pre-existing TSRD diagnosis; for the remaining 98.5% that did not, this predictor was not very informative. This is consistent with the results of the benchmark model that used pre-existing TSRD as the sole predictor, which performed poorly (AUC = 0.56). This suggests that all variables in the model were weak as individual predictors, and that the final model’s predictive power arised from the collective contribution of variables and their interactions. The SHAP plot also provides information about the potential directionality of impact on predictions (note that the GBM is a nonparametric model that can capture non-linear relations). For most predictors, higher values (indicated in blue) were associated with higher predicted probability of TSRD, whereas lower values (indicated in yellow) were associated with a lower predicted probability of TSRD. Two clear exceptions to this pattern were age and heart rate. Table 3 contains a complete list of core predictors which cut across nine domains, including pre-existing TSRD diagnosis, other mental health diagnoses, patient characteristics, medications, lab values, vital signs, medical comorbidity, mental status, and admissions data. Weighted descriptive statistics are provided for the development and test samples, including the median and interquartile range (IQR) of each continuous predictor and proportions for the binary predictors. The standardized total- and net-predictive values from the core predictor analysis are also provided; these reflect the strength of each predictor’s association with the outcome, and its unique contribution to the prediction of the outcome, respectively.

**Table 3:** Descriptive statistics and total and net information (scaled) of the 29 core predictors in the final model.

Predictor	Domain	Development Sample, Median [IQR] or N (%)	Test Sample, Median [IQR] or N (%)	Total Information	Net Information
Pre-existing trauma- or stressor-related disorder (diagnosis present)	mental health diagnosis	3307 (1.5%)	1963 (1.6%)	1	1
Age (years)	demographics	71 [25]	70 [25]	0.064	0.028
Psychotherapeutic medication class (number of administrations)	medication	0 [3]	0 [3]	0.041	0.071
Comorbidity score	other admission data	43 [80]	48 [84]	0.032	0.048
Pre-existing mood disorder (diagnosis present)	mental health diagnosis	30,917 (14.2%)	16,421 (13.2%)	0.028	0.010
Oxygen saturation (mean value)	vital sign	96.94 [2.10]	96.85 [2.11]	0.024	0.018
White blood cell count (first value)	lab value	10.8 [7.20]	11 [7.30]	0.024	0.032
Systolic blood pressure (first value)	lab value	133 [35]	133 [35]	0.023	0.059
White blood cell count (last value)	lab value	8.30 [4.60]	8.30 [4.60]	0.023	0.033
Benzodiazepine (number of administrations)	medication	0 [0]	0 [0]	0.022	0.028
Heart rate (mean value)	vital sign	82.78 [18.14]	83.15 [18.27]	0.020	0.019
Pre-existing anxiety disorder (diagnosis present)	mental health diagnosis	18,218 (8.4%)	10,068 (8.1%)	0.020	0.025
Analgesic (number of administrations)	medication	4 [11]	3 [10]	0.019	0.014
Creatinine (first value)	lab value	0.97 [0.68]	1.00 [0.69]	0.017	0.022
Lactate (mean value)	lab value	1.40 [0.77]	1.50 [0.8]	0.017	0.017
Alert and oriented X3	mental status	179,393 (87.2%)	98,105 (87.2%)	0.015	0.046
Bicarbonate (first value)	lab value	25 [5]	26 [5]	0.015	0.019
Diastolic blood pressure (mean value)	vital sign	67.41 [12.44]	67.46 [12.96]	0.015	0.010
Length of stay (days)	hospitalization	3.10 [3.60]	3.00 [3.30]	0.014	0.030
Temperature (first value)	vital sign	98.40 [1.40]	98.40 [1.40]	0.014	0.043
Analgesic, opioid agonist (number of administrations)	medication	0 [3]	0 [2]	0.013	0.016
Analgesic, opioid (number of administrations)	medication	0 [4]	0 [3]	0.013	0.034
Arterial PaO_2_ (first value)	lab value	80 [48]	81 [47]	0.013	0.030
Hematocrit (maximum value)	lab value	37.50 [7.80]	37.70 [8.30]	0.013	0.017
Lactate (minimum value)	lab value	1.10 [0.60]	1.20 [0.70]	0.013	0.033
Height (inches)	other admission data	66.00 [6.01]	66.00 [6.01]	0.012	0.018
Analgesic, nonsteroidal anti-inflammatory (number of administrations)	medication	0 [0]	0 [0]	0.011	0.041
Sex (female)	demographics	117,768 (54.2%)	66,180 (53.1%)	0.011	0.023
Olanzapine (number of administrations)	medication	3 [6]	4 [5]	0.010	0.027

*Note*. Total information denotes the relative contribution of predictors through direct associations with the outcome, and net information denotes the relative unique information contributed. Core predictors were identified using gradient boosting machines (GBM) 20% of the development sample (*n* = 29,822 hospitalizations). The remaining 80% of the development sample (*n* = 118,559 hospitalizations) was used to develop a GBM with only these 29 core predictors (selected as the optimal model).

Fifteen predictors (51.7%) had missing data. Arterial PaO2 and lactate are lab values that are not necessarily collected for every patient. Accordingly, 84.6% of the development sample (86.0% in the test sample) did not have Arterial PaO_2_ data, and 30.6% of both the development and test sample did not have lactate data. The remaining lab and vital sign values (bicarbonate, creatinine, white blood cell count, temperature, systolic blood pressure, hematocrit, diastolic blood pressure, heart rate, oxygen saturation), and mental status (alert and oriented X 3) were missing for 5.3–5.9% of the development sample and 9.7–10.1% of the test sample. The only remaining variable with missing data was height (0.5% in the development sample, and 0.4% in the test sample). These were missing due to the application of preprocessing steps in the data extraction that remove typos and clinically unrealistic values (e.g., negative values, extreme values). As noted in the Methods, all of the modeling approaches dealt with missing predictor data so that all hospitalizations could be included in the analysis. ROC curves and corresponding AUC statistics were nearly identical in the subsample of participants who had or did not have missing predictor data related to typos or clinically unrealistic values (Supplementary Fig. 3).

**Fig. 3:**
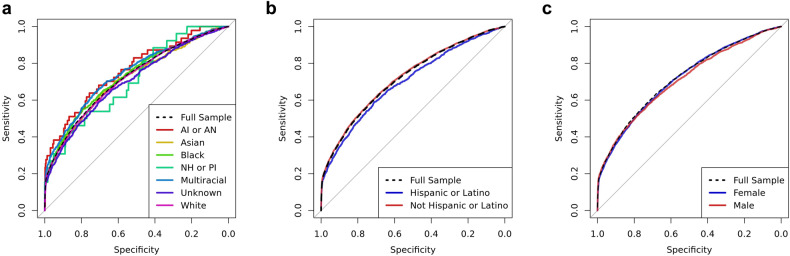
Receiver operating characteristic curves of model performance across sociodemographic subgroups in the independent test sample. **a** ROC curves by self-reported race (AI or AN = American Indian or Alaska Native; NH or PI = Native Hawaiian or Pacific Islander; Multiracial = more than one category endorsed; Unknown = racial category not recorded). **b** ROC curves by self-reported ethnicity. **c** ROC curves by self-reported sex.

Model performance in the independent test sample was also examined across the self-reported demographic characteristics of ethnicity, race, and sex. Across these subgroups, TSRD prevalence ranged from 4.8% among the Asian subgroup to 10.1% among the Black or African American subgroup. Mean classification performance across subgroups, indexed by AUC, ranged from 0.70 to 0.76 (Supplementary Table 2). Figure 3 displays the ROC curves for subgroups across ethnicity, race, and sex, which showed considerable overlap in sensitivity across different levels of specificity. This suggests that model performance (in terms of sensitivity and specificity) is comparable across subgroups at a wide range of potential thresholds.

## Discussion

Our study makes several contributions to the literature on prediction models of trauma-related psychiatric outcomes developed in hospital settings. This is the first study to focus on the life-threatening trauma of sepsis, a leading cause of death among hospitalized individuals [1]. Our sample size is over 300 times larger than the largest prior study [11]; this facilitated the comparison of model performance across sociodemographic subgroups, which is a critical step in ensuring that models can be implemented fairly across diverse patients. Our only exclusion criterion was death during the hospitalization, which may increase generalizability by ensuring that the model development and validation process included patients with conditions that often form part of exclusion criteria in the prior studies. In contrast to the use of a random sample for model validation, we used a more rigorous temporal validation approach which is more closely aligned to the goal of estimating model performance in future cases. While the AUC of our model (0.72) was in the lower end of the range of performance measured in previous models (AUC range 0.71 to 0.89) [7–12], our model is the first to solely rely on data from the electronic health records for predictors and outcomes. This increases the feasibility of model implementation because our model does not add any assessment burden to clinicians or patients. Together, this highlights the clinical significance of our results, which suggest that risk for TSRD after survival from sepsis can be stratified by our model. These findings are relevant to real-world clinicians because they indicate potential for a tool that may augment decision-making around prevention and early intervention for TSRD without increasing assessment burden during the hospitalization.

Variable importance analyses should not be causally interpreted, but they provide several insights about the prediction model. First, it is not surprising that the final set of predictors cut across a wide range of domains given that the core predictors approach is designed to identify variables that make unique contributions to the prediction. In fact, the only category of predictors that was not used by the optimal model was the clinician note variables. Although it is plausible the type and severity of presenting symptoms captured in the clinician notes are useful predictors of TSRD, this information may have been also captured by other variables that are associated with sepsis severity, including labs, vital signs, and medications administered. It is also possible that the NLP-identified symptoms were less consistent as predictors due to clinician variability in recording practices. Second, despite its status as one of the strongest predictors, pre-existing TSRD diagnosis on its own was a poor predictor of post-discharge TSRD. This highlights a strength of the GBM algorithm, which can make good predictions from individually weak predictors by capturing complex, high-dimensional interactions.

Our use of real-world EHR data resulted in a pool of predictors that have not been examined in previous predictive models of trauma-related psychiatric outcomes after hospitalization. Nevertheless, 10 of the 29 predictors in our model overlap with key predictors in previous studies. For example, all of the previous studies we reviewed (which mostly focused on PTSD outcomes) found that a measure of PTSD sequelae in the hospital (e.g., pre-existing condition, symptom levels from previous trauma, acute PTSD symptoms in the ED) was a strong predictor of PTSD in the follow-up phase [7–12]. Moreover, the studies that tested benchmark models similar to ours also found poorer performance relative to complex models with more predictors, with most AUCs in the 0.56 to 0.66 range [8–11], and one smaller study finding an AUC in the good range (0.78) [12]. Most of the reviewed studies also found age to be a key predictor [7–10, 12]; however, the direction of the association has been mixed, which highlights the need for caution when interpreting results at the level of individual predictors. The remaining predictors in our model that overlap with predictors identified in other studies were pre-existing mood disorder [8, 9, 11, 12], heart rate [7, 9, 11, 12], systolic blood pressure [7, 8], mental status [10, 12], length of stay [10, 12], pre-existing anxiety disorder [12], creatinine [8], and hematocrit [8]. Together this suggests that the predictive power of our model, as well as prior models, arises from a combination of information related to mental health (e.g., pre-existing diagnoses, psychiatric medications) alongside variables that capture the severity of the acute trauma (e.g., lab values, length of stay, administration of analgesics). Although none of these predictive models were designed to test causal hypotheses, our study replicated the predictive value of the aforementioned variables and also identified novel predictors, which is an important contribution to improving predictive models for trauma-related psychiatric outcomes. Future studies that include patients with other conditions in addition to sepsis can determine whether some of the predictors we found are unique to sepsis patients.

We note several limitations. We performed an exhaustive search for TSRD diagnostic codes across all healthcare system encounters in the year post-discharge; however, it is possible that some patients did not report symptoms to their providers and were therefore not assessed for TSRD. Although several algorithms have been developed and tested within VA healthcare systems to identify PTSD cases using a combination of diagnostic codes and other EHR data [38, 39], future research is necessary to improve the identification of TSRDs in civilian healthcare systems. This may improve the identification of cases that may otherwise go undetected, including individuals with subthreshold symptoms of PTSD, which have been linked to clinically significant distress and impairment [40]. Additionally, future studies based on real-world data would benefit from large-scale implementation of self-report assessments of TSRD symptoms in order to improve the identification of positive (and negative) cases, given that there are differences in access to care across patients, and diagnostic practices across clinicians. This would also provide an avenue to assess whether other trauma besides (or in addition to) the sepsis hospitalization played a role in the diagnosis. To our knowledge, this is the first prediction model for TSRD that incorporated natural language predictors from clinician notes. Nevertheless, a limitation is that we focused on clinician notes from the first 24 hours of hospitalization in order to capture signs and symptoms of the presenting illness. Future studies can incorporate notes from the complete hospitalization period, and apply recent advances in natural language processing to extract information. Although we found comparable classification performance across sociodemographic subgroups, algorithmic fairness is a highly active area of research and there is currently no consensus on the optimal criteria to assess fairness [37]. Moreover, we selected the sociodemographic characteristics that are most commonly examined and that are well characterized in the EHR; future research can examine additional factors, including social determinants of health, which are increasingly integrated into EHR. Although we used inverse probability weighting to reduce the potential for attrition bias in performance estimates, unmeasured confounding is a possibility. Predictive models are not designed to test causal hypotheses; therefore, the core predictors identified during the model development phase should not be interpreted as causal factors [41]. Additionally, predictors that were not selected by the core-predictor analysis should not be assumed to be unrelated to the outcome. Finally, a key strength of this study is the minimal exclusion criteria; although we excluded individuals with lapses in health insurance coverage, we did not impose criteria related to the number of encounters with the healthcare system during the follow-up window, which may have associations with the presence of TSRD diagnosis in the EHR.

## Conclusions

The predictive model we developed demonstrates that risk for a TSRD diagnosis in the year after survival from sepsis can be stratified without additional assessment burden on clinicians and patients because it relies on a parsimonious set of predictors that are a standard part of hospital workflows. Future research is needed to clarify whether our model attains a level of accuracy needed for cost-effective implementation of targeted prevention and early intervention strategies.

### Supplementary information

Supplemental Material

## Data Availability

The data from this study cannot be shared publicly as we do not have permission from patients to share their data outside of the Kaiser Permanente Northern California healthcare system.
