# Supplementary material for: Development and validation of a machine learning model using electronic health records to predict trauma- and stressor-related psychiatric disorders after hospitalization with sepsis
Source: Transl Psychiatry. 2023 Dec 18;13:400. doi: 10.1038/s41398-023-02699-6 (PMC10730505; doi:10.1038/s41398-023-02699-6)
Supplement: Supplementary file 1 — Supplemental Material [file 41398_2023_2699_MOESM1_ESM.docx]

Supplementary Information for *Development and validation of a machine learning model using electronic health records to predict trauma- and stress-related psychiatric disorders after hospitalization with sepsis*

**Table of Contents**

1. Supplementary Table 1. Diagnostic codes used to establish presence of a trauma- or stress-related disorder in the 12-months post-discharge
2. Supplementary Table 2. Outcome prevalence and performance metrics across sociodemographic subgroups in the independent test sample.
3. Supplementary Fig. 1: Schematic of modeling pipeline
4. Supplementary Fig. 2: Model performance after removal of encounters from patients who also had encounters in the development sample.
5. Supplementary Fig. 3: Receiver operating characteristics curve and corresponding AUC statistic of subsets of the test sample with and without missing predictor data.
6. Appendix 1. TRIPOD Checklist: Prediction Model Development and Validation

Supplementary Table 1. Diagnostic codes used to establish presence of a trauma- or stress-related disorder in the 12-months post-discharge

| **Diagnosis** | **Code** |
| --- | --- |
| Posttraumatic stress disorder | ICD 9: 309.81; ICD 10: F43.10, F43.11, F43.12 |
| Acute stress disorder | ICD 9: 308.9; ICD 10: F43.0 |
| Adjustment disorder | ICD 9: 309.0, 309.1, 309.24, 309.28, 309.29, 309.3, 309.4; ICD 10: F43.20, F43.21, F43.22, F43.23, F43.24, F43.25, F43.29 |
| Other Specified Trauma- and Stressor-Related Disorder | ICD 9: 309.89; ICD 10: F43.8 |
| Unspecified Trauma- and Stressor-Related Disorder | ICD 9: 309.9; ICD 10: F43.9 |

Supplementary Table 2. Outcome prevalence and performance metrics across sociodemographic subgroups in the independent test sample.

|  | N (%) | Prevalence [95% CI] | AUC  [95% CI] |
| --- | --- | --- | --- |
| Full Sample | 128,783 (100%) | 7.4 [7.3, 7.6] | 0.72 [0.71, 0.72] |
| Ethnicity (self-reported) |  |  |  |
| Hispanic or Latino | 20,903 (16.2%) | 7.8 [7.3, 8.3] | 0.70 [0.68, 0.72] |
| not Hispanic or Latino | 107,880 (83.8%) | 7.3 [7.2, 7.5] | 0.72 [0.71, 0.73] |
| Race (self-reported) |  |  |  |
| American Indian or Alaska Native | 803 (0.6%) | 9.3 [6.8, 12.0] | 0.76 [0.69, 0.83] |
| Asian | 14,252 (11.1%) | 4.8 [4.3, 5.2] | 0.70 [0.68, 0.73] |
| Black | 13,407 (10.4%) | 10.1 [9.4, 10.6] | 0.72 [0.70, 0.74] |
| Multiracial | 8,633 (6.7%) | 7.7 [7.0, 8.4] | 0.76 [0.73, 0.78] |
| Native Hawaiian or Pacific Islander | 843 (0.7%) | 6.0 [4.1, 8.0] | 0.74 [0.65, 0.83] |
| Unknown | 16,123 (12.5%) | 7.2 [6.7, 7.7] | 0.70 [0.67, 0.72] |
| White | 74,722 (58.0%) | 7.5 [7.3, 7.7] | 0.71 [0.70, 0.72] |
| Sex (self-reported) |  |  |  |
| female | 67,924 (52.7%) | 8.4 [8.1, 8.6] | 0.72 [0.71, 0.73] |
| male | 60,859 (47.3%) | 6.4 [6.1, 6.6] | 0.70 [0.69, 0.71] |

Supplementary Fig. 1: Schematic of modeling pipeline


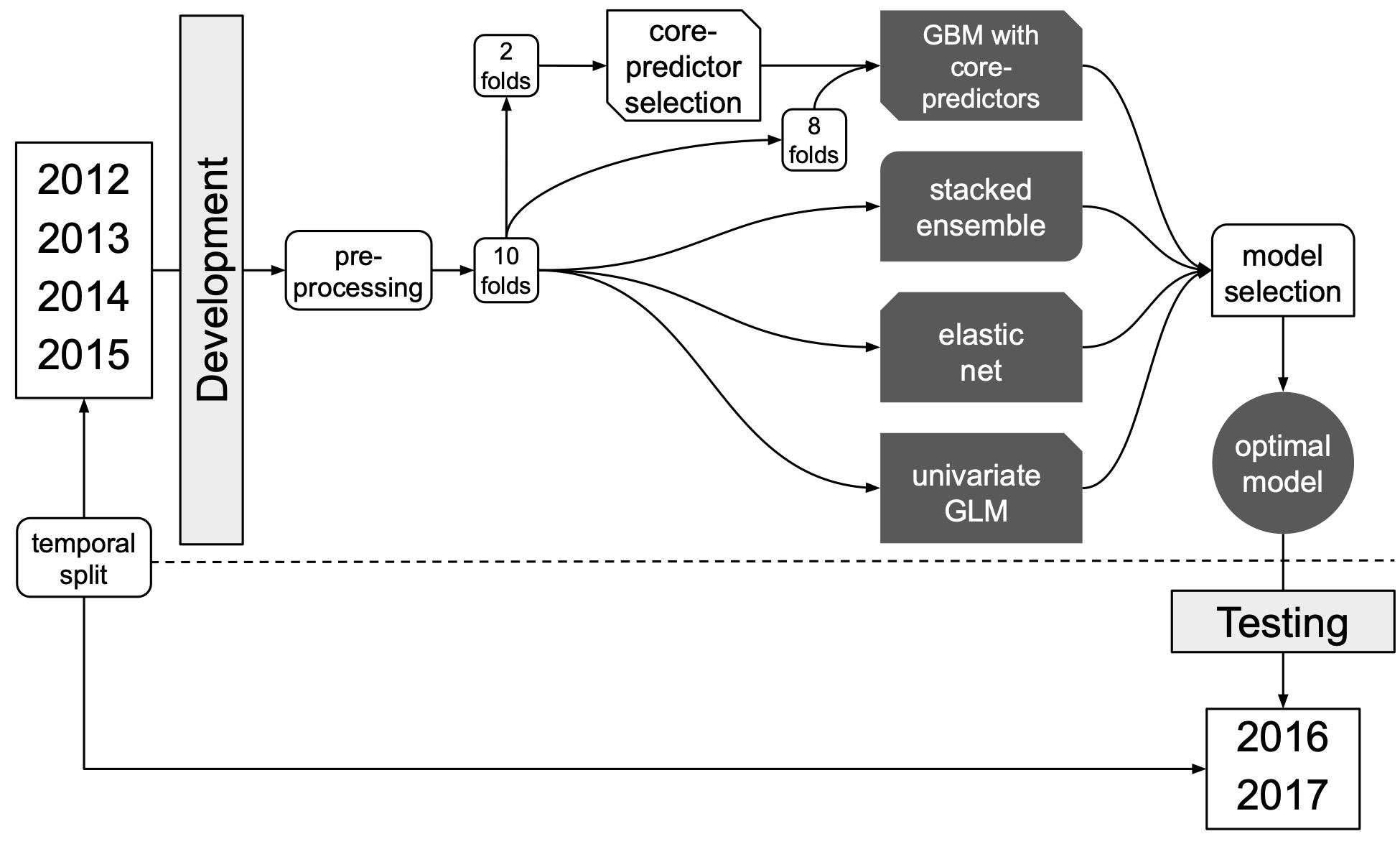


Supplementary Fig. 2: Model performance after removal of encounters from patients who also had encounters in the development sample.

**
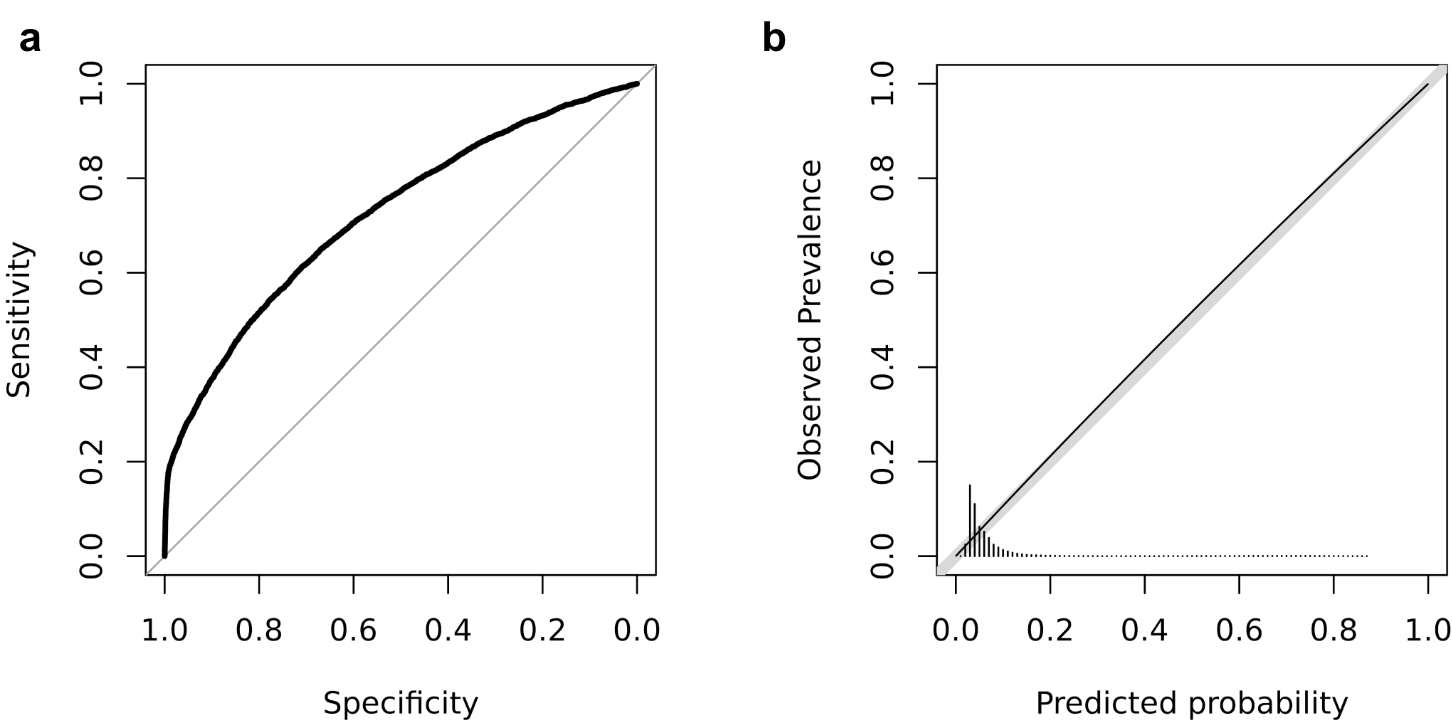
**

**a,** Receiver operating characteristics (ROC) curve illustrating the tradeoff between model sensitivity and specificity based on mode-predicted probabilities of TSRD in the independent test set. **b,** Logistic calibration curve illustrating the correspondence between model-predicted probabilities and observed prevalence of TSRD in the independent test set.

Supplementary Fig. 3: Receiver operating characteristics curve and corresponding AUC statistic of subsets of the test sample with and without missing predictor data.


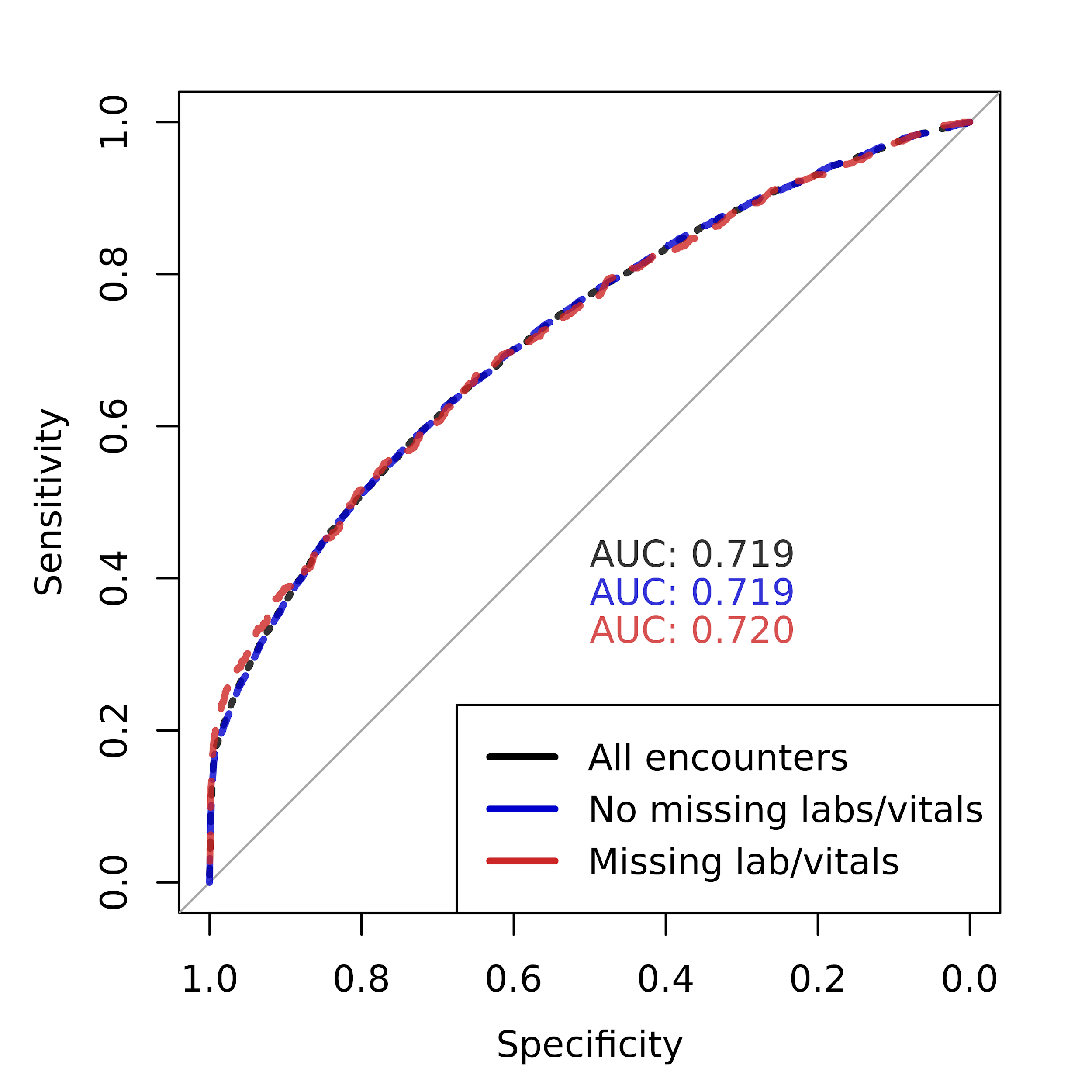


Approximately 11.0% of the sample had missing data on predictors related to bicarbonate, creatinine, white blood cell count, temperature, systolic blood pressure, hematocrit, diastolic blood pressure, heart rate, oxygen saturation, mental status, and/or height. Performance on this subset (red) is compared to performance of the full sample (black) and the subset with no missing data on the aforementioned predictors (blue).

Appendix 1. TRIPOD Checklist: Prediction Model Development and Validation

| **Section/Topic** | **Item** |  | **Checklist Item** | **Page** |
| --- | --- | --- | --- | --- |
| **Title and abstract** | | | | |
| Title | 1 | D;V | Identify the study as developing and/or validating a multivariable prediction model, the target population, and the outcome to be predicted. | 1 |
| Abstract | 2 | D;V | Provide a summary of objectives, study design, setting, participants, sample size, predictors, outcome, statistical analysis, results, and conclusions. | 2 |
| **Introduction** | | | | |
| Background and objectives | 3a | D;V | Explain the medical context (including whether diagnostic or prognostic) and rationale for developing or validating the multivariable prediction model, including references to existing models. | 3 |
|  | 3b | D;V | Specify the objectives, including whether the study describes the development or validation of the model or both. | 3-4 |
| **Methods** | | | | |
| Source of data | 4a | D;V | Describe the study design or source of data (e.g., randomized trial, cohort, or registry data), separately for the development and validation data sets, if applicable. | 4 |
|  | 4b | D;V | Specify the key study dates, including start of accrual; end of accrual; and, if applicable, end of follow-up. | 4 |
| Participants | 5a | D;V | Specify key elements of the study setting (e.g., primary care, secondary care, general population) including number and location of centres. | 4 |
|  | 5b | D;V | Describe eligibility criteria for participants. | 4 |
|  | 5c | D;V | Give details of treatments received, if relevant. | NA |
| Outcome | 6a | D;V | Clearly define the outcome that is predicted by the prediction model, including how and when assessed. | 7, Supp. Table 1 |
|  | 6b | D;V | Report any actions to blind assessment of the outcome to be predicted. | NA |
| Predictors | 7a | D;V | Clearly define all predictors used in developing or validating the multivariable prediction model, including how and when they were measured. | 4-7 |
|  | 7b | D;V | Report any actions to blind assessment of predictors for the outcome and other predictors. | NA |
| Sample size | 8 | D;V | Explain how the study size was arrived at. | 4, 10, Fig. 1 |
| Missing data | 9 | D;V | Describe how missing data were handled (e.g., complete-case analysis, single imputation, multiple imputation) with details of any imputation method. | 4, 7 |
| Statistical analysis methods | 10a | D | Describe how predictors were handled in the analyses. | 4-7 |
|  | 10b | D | Specify type of model, all model-building procedures (including any predictor selection), and method for internal validation. | 7-9 |
|  | 10c | V | For validation, describe how the predictions were calculated. | 8-9 |
|  | 10d | D;V | Specify all measures used to assess model performance and, if relevant, to compare multiple models. | 8-9 |
|  | 10e | V | Describe any model updating (e.g., recalibration) arising from the validation, if done. | NA |
| Risk groups | 11 | D;V | Provide details on how risk groups were created, if done. | 9 |
| Development vs. validation | 12 | V | For validation, identify any differences from the development data in setting, eligibility criteria, outcome, and predictors. | 4, 9, Table 1 |
| **Results** | | | | |
| Participants | 13a | D;V | Describe the flow of participants through the study, including the number of participants with and without the outcome and, if applicable, a summary of the follow-up time. A diagram may be helpful. | Fig. 1 |
|  | 13b | D;V | Describe the characteristics of the participants (basic demographics, clinical features, available predictors), including the number of participants with missing data for predictors and outcome. | Fig. 1; Table 1 |
|  | 13c | V | For validation, show a comparison with the development data of the distribution of important variables (demographics, predictors and outcome). | 9, Table 1 |
| Model development | 14a | D | Specify the number of participants and outcome events in each analysis. | 9 |
|  | 14b | D | If done, report the unadjusted association between each candidate predictor and outcome. | NA |
| Model specification | 15a | D | Present the full prediction model to allow predictions for individuals (i.e., all regression coefficients, and model intercept or baseline survival at a given time point). | NA |
|  | 15b | D | Explain how to use the prediction model. | NA |
| Model performance | 16 | D;V | Report performance measures (with CIs) for the prediction model. | 9, Fig. 2, Fig. 3, Table 2, Table 3 |
| Model-updating | 17 | V | If done, report the results from any model updating (i.e., model specification, model performance). | NA |
| **Discussion** | | | | |
| Limitations | 18 | D;V | Discuss any limitations of the study (such as nonrepresentative sample, few events per predictor, missing data). | 12 |
| Interpretation | 19a | V | For validation, discuss the results with reference to performance in the development data, and any other validation data. | 11 |
|  | 19b | D;V | Give an overall interpretation of the results, considering objectives, limitations, results from similar studies, and other relevant evidence. | 11-13 |
| Implications | 20 | D;V | Discuss the potential clinical use of the model and implications for future research. | 13 |
| **Other information** | | | | |
| Supplementary information | 21 | D;V | Provide information about the availability of supplementary resources, such as study protocol, Web calculator, and data sets. | Supp. Material |
| Funding | 22 | D;V | Give the source of funding and the role of the funders for the present study. | Acknowledgements |

*Items relevant only to the development of a prediction model are denoted by D, items relating solely to a validation of a prediction model are denoted by V, and items relating to both are denoted D;V. We recommend using the TRIPOD Checklist in conjunction with the TRIPOD Explanation and Elaboration document.
